# Context-Specific Outdoor Time and Physical Activity among School-Children Across Gender and Age: Using Accelerometers and GPS to Advance Methods

**DOI:** 10.3389/fpubh.2014.00020

**Published:** 2014-03-11

**Authors:** Charlotte Demant Klinker, Jasper Schipperijn, Jacqueline Kerr, Annette Kjær Ersbøll, Jens Troelsen

**Affiliations:** ^1^Department of Sports Science and Clinical Biomechanics, University of Southern Denmark, Odense, Denmark; ^2^Department of Family and Preventive Medicine, University of California San Diego, San Diego, CA, USA; ^3^The National Institute of Public Health, University of Southern Denmark, Copenhagen, Denmark

**Keywords:** children, physical activity, accelerometer, GPS, spatial behavior, context-specific, outdoor behavior

## Abstract

**Introduction:** Being outdoors has a positive influence on health among children. Evidence in this area is limited and many studies have used self-reported measures. Objective context-specific assessment of physical activity patterns and correlates, such as outdoor time, may progress this field.

**Aims:** To employ novel objective measures to assess age and gender differences in context-specific outdoor weekday behavior patterns among school-children [outdoor time and outdoor moderate to vigorous physical activity (MVPA)] and to investigate associations between context-specific outdoor time and MVPA.

**Methods:** A total of 170 children had at least one weekday of 9 h combined accelerometer and global positioning system data and were included in the analyses. The data were processed using the personal activity and location measurement system (PALMS) and a purpose-built PostgreSQL database resulting in context-specific measures for outdoor time, outdoor MVPA, and overall daily MVPA. In addition, 4 domains (leisure, school, transport, and home) and 11 subdomains (e.g., urban green space and sports facilities) were created and assessed. Multilevel analyses provided results on age and gender differences and the association between outdoor time and MVPA.

**Results:** Girls compared to boys had fewer outdoor minutes (*p* < 0.05), spent a smaller proportion of their overall daily time outdoors (*p* < 0.05), had fewer outdoor MVPA minutes during the day (*p* < 0.001) and in 11 contexts. Children compared to adolescents had more outdoor minutes (*p* < 0.05). During school and within recess, children compared to adolescents had more outdoor MVPA (*p* < 0.001) and outdoor time (*p* < 0.001). A 1-h increase in outdoor time was associated with 9.9 more minutes of MVPA (*p* < 0.001).

**Conclusion:** A new methodology to assess the context-specific outdoor time and physical activity patterns has been developed and can be expanded to other populations. Different context-specific patterns were found for gender and age, suggesting different strategies may be needed to promote physical activity.

## Introduction

Being outdoors, as opposite to being indoors, may have a positive influence on a range of health parameters among children and adolescents (1). Being outdoors has also been identified as a correlate for more active play (2), enhanced physical activity levels (3–5), lower prevalence of overweight (6), and independent mobility (2). Being outdoors may help children and adolescents to reach 60 daily minutes of moderate to vigorous physical activity (MVPA); a minimum level recommended for children under the age of 18 by the World Health Organization and many national health authorities (7). Sustained low levels of physical activity are seen in many countries (8, 9), and often a decline in physical activity in the transition from childhood to adulthood is reported (10, 11).

Effective interventions or policies are needed to promote physical activity, and ecological models underpinning the importance of an active living lifestyle and built environmental influences, have received widespread recognition (12). The built environment consists of neighborhoods, roads, buildings, food sources, and recreational facilities: the places in which people live, work, are educated, eat, and play (13). If outdoor time is shown to be important for physical activity, policies to provide safe outdoor environments may be warranted. Evidence in this area is still limited and mixed as many studies on the association between the built environment and physical activity have used cross-sectional study designs and relied on self-reported data or daily averages from objective data. The association between the built environment and physical activity seems to be highly context-specific (4, 5), and inconsistencies in correlate studies may be partly explained by some studies measuring overall physical activity and not context-specific physical activity (e.g., physical activity during active transport, activity in urban green space or at playgrounds). An even better precision and perhaps correlation may be obtained if *physical activity patterns* are assessed (14–17) meaning that daily physical activity is assessed in different context throughout the day. Context-specific knowledge of physical activity patterns and correlates, such as outdoor time, may be a way to progress this field. Due to methodological challenges, this association has rarely been investigated using objective measures.

A valuable tool for improving the assessment of physical activity and outdoor behavior is the global positioning system (GPS). The GPS is a satellite-based global navigation system that provides a precise location at any point on the surface of Earth based on the position of satellites in the sky. The development of lightweight, affordable, and portable GPS receivers that can log individuals’ locations continuously during consecutive days means that they can be used to objectively assess context-specific behavior. With the rapid development of the market for GPSs the battery life, memory capacity, and precision are improving (18). When combined with a device measuring physical activity, such as an accelerometer, it becomes possible to accurately assess patterns of context-specific physical activity (15–17, 19–24). The GPS receivers also collect information on the number of satellites used by and in view of the GPS receiver that can be used to provide estimates for outdoor times, and if assessed, outdoor physical activity (19, 20, 25, 26). A feasibility study by Tandon and colleagues among pre-schoolers concluded that it was feasible and valid to use a Qstarz GPS to distinguish indoor and outdoor time when using the personal activity and location measurement system (PALMS) (27) to process the data (26). Furthermore, information on speed and distance traveled can be used to assess mode of transport (28, 29). At the moment, new evidence about context-specific physical activity behaviors is being generated on the basis of combined accelerometer and GPS data and this paper is part of a developing research area.

The aim of this paper is to employ objective measures to assess the context-specific outdoor weekday pattern among school-children and determine which contexts contribute to most outdoor time. Furthermore, the aim is to assess the contexts where weekday outdoor MVPA occurs and investigate how much of total daily MVPA is outdoors. As gender is a strong correlate for physical activity, and age a probable correlate (3), age and gender differences were assessed for these two aims. Finally, the aim was to investigate the association between context-specific outdoor time and MVPA.

## Materials and Methods

### Participants and procedures

Children in grade 5–8 (11–16 years old) were recruited from four schools, participating in the When Cities Move Children (WCMC) study. The WCMC study is a natural experiment conducted in a deprived neighborhood in Copenhagen, the capital of Denmark, evaluating how major improvements to the built environment influences physical activity and movement patterns. There are 9300 people living in the district; children comprise 20% of the population, and almost 70% of the children are immigrants or descendants of immigrants (30). Only baseline data collected in 2010–2011 were employed for the current analyses conducted in summer 2013. We chose to sample participants in a time period where it was hypothesized that the average day length, temperature, and rain were comparable in a Danish context. Data from three schools were collected in spring while data from the fourth school were collected in fall, corresponding to 85% of the data collected in spring and 15% in fall. There were no differences between participants from these two seasons by gender, age, BMI, MVPA or combined accelerometer, and GPS wear time.

Eligible children (*N* = 623) and their parents received personalized information about the nature and procedures of the study in Danish and if needed in one of four other languages (Arabic, Somali, Turkey, and Urdu) to match the ethnic background of the parents. The parents and children were notified that participation was voluntary and that they could withdraw at any stage. A passive informed consent procedure was used, where students were included unless the parents withdrew consent as this procedure has been found to be ethically appropriate in low-risk research in adolescents (31). The Danish Ethical Regional Committee reviewed the study protocol and concluded that formal ethics approval was not required. The study is registered and approved by the Danish Data Protection Agency (reference number: 2009-41-3943). Consent was obtained from 523 children and there were no overall differences between responders and non-responders by gender, ethnicity, BMI, or parental work status. However, the non-response was unequally distributed by age and school with the drop-out being greater among adolescents (children 11.7%, adolescents 21.4%, *p* = 0.001) and in two schools (*p* < 0.001).

**Figure 1:**
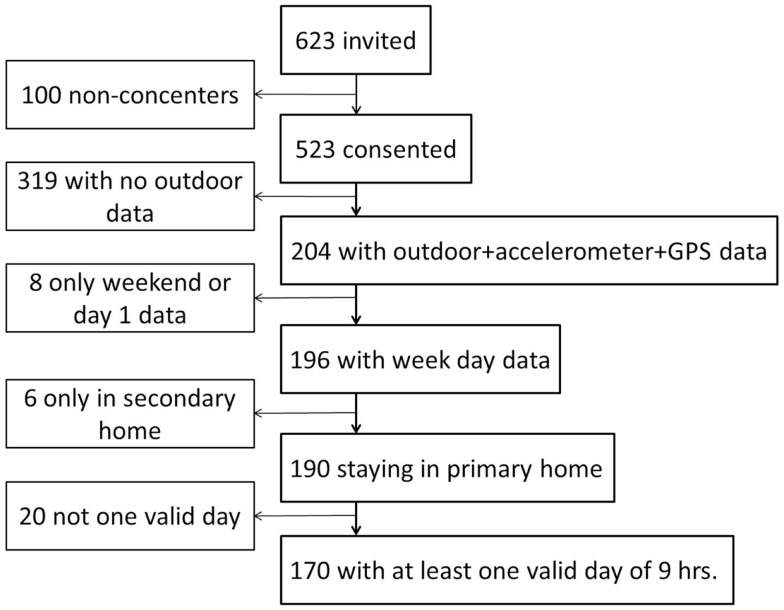
**Reduction of the study population**.

The inclusion criteria were at least one valid weekday of 9 h combined accelerometer and GPS wear time, excluding day 1 data, weekend data, participants not staying in their primary home during data collection (for participants with divorced parents), and participants who did not have any outdoor data. Data from day 1 were removed as the equipment was fitted at different times during the day, leaving the participants with unequal opportunities to obtain enough hours to become a valid day. Furthermore, the analyses conducted required a full day behavior pattern. Weekend data were removed as weekday data provided the greatest variability in domains and subdomains and because weekends and weekdays are not directly comparable in terms of domains/subdomains, i.e., children do not attend school on weekends. Data from children with divorced parents who stayed in their secondary home during data collection were removed as only the primary parent’s address was known to be located within the assessed neighborhood. Due to a software problem with the initializing system used to set-up the GPS devices, almost all GPS on two schools did not record the satellite signal to noise ratio (SNR), which meant that the time spent outdoors could not be assessed. The majority of participants from these two schools were excluded, leaving 204 participants with combined accelerometer and GPS data and outdoor time measures. Eight participants were excluded as they only had weekend data or data from day 1, 6 participants were excluded as they only were in their secondary home during data collection, and 20 were excluded as they did not have one valid day of 9 h of combined weekday data. This led to a total sample for this study of 170 participants out of the 523 consenters (32.5%). There were no differences in background characteristics, such as gender, BMI, parental employment status, and immigrant status, between those who provided complete measures (*n* = 170) and those who were excluded (*n* = 353). However, the drop-out was unequally distributed by school (*p* < 0.001) and age (*p* < 0.001). Figure 1 displays a flow diagram of the reduction of the population.

### Physical activity – accelerometer measures

Objective physical activity levels were assessed using the tri-axial Actigraph GT3X accelerometer during seven consecutive days. On day 8, the participants handed in the equipment. Only the vertical axis was used for this study. The accelerometer has the ability to yield measures of volume, frequency, intensity, and duration of children’s physical activity (32). Several reviews have concluded that accelerometers provide an accurate, reliable, and practical objective measure of physical activity in children and adolescents (33, 34). The data were recorded at 2 s epochs. All students were instructed to wear the monitor on their hip during waking hours and to take it off only for showering, bathing, or any water sports. They were asked to record in a diary the times they took the monitor off and the reason for doing so. Data from the returned monitors were downloaded using the ActiLife 4.4.1 software and screened for file size to detect potential equipment or download problems.

### Contexts – GPS measures

QStarz BT-Q1000X GPS units were used to record movement. The QStarz unit has shown relatively high accuracy across a range of sites (e.g., canopy and open sky) and good inter-unit reliability compared to other units (18, 29, 35). The GPS units were set-up using BT747 open source software (bt747.org). Units were configured to log data every 15 s, a compromise between optimal frequency (i.e., 2 s as the accelerometer) and data storage capacity of the units over a 7-day period. The units were set to stop logging when the memory was full and to record: date, time, longitude and latitude (used to calculate location), elevation, speed (used to calculate transport mode), and the number of satellites in view and used (used to calculate the Signal-to-Noise-Ratio (SNR)). SNR’s can be used to estimate if the GPS is outdoors. After set-up, the fully charged GPS were turned off. On day 1 of data collection the research team turned the GPS units on, and taped the on/off button to prevent it from sliding to off. The children were instructed to leave the tape on, and *not* turn the unit off during data collection. They were instructed to wear the GPS on the same belt as the accelerometer but on the opposite hip, and only to take it off as instructed with the accelerometer. After data collection, the data were downloaded using bt747 software and screened for file size to detect the potential equipment or download problems.

Participants who lost or had malfunctioning devices during data collection, and informed the research team, had their device exchanged, and their data were later merged into one data-file. A total of 14 participants lost one or both devices, or returned malfunctioning devices when data collection was complete.

### Correlates

Children’s immigrant status was obtained from Statistics Denmark using a unique personal identification number assigned to all people in Denmark (36) and children were categorized as Danish versus immigrant (not born in Denmark) or descendent (born in Denmark but parents not born in Denmark). Parental employment status was obtained from Danish registers on personal labor market affiliation (37) and categorized as parents working versus one or both unemployed. Age was dichotomized into grade 5–6 (age 11–13) versus grade 7–8 (13–16), to best approximate children and adolescents. Information on self-reported height and weight was obtained from a questionnaire (E-survey) completed during data collection. BMI was calculated using Cole’s age and gender specific cut off points (38) and included as a continuous variable.

### Steps taking to increase validity

Questionnaire data were used to assess if children living with divorced parents were residing in their primary home (i.e., the local neighborhood) during the data collection period. Participants completed a daily diary during data collection to assess non-wear and changes to the ordinary school schedule. Students were asked to note school (non-)attendance times, and if it had been a regular school day (if not, why not?). The schools furthermore provided detailed class timetables for the data collection period including information on start and end of school days, recess and physical education (PE). These measures were used to adjust and improve the quality of the combined GPS and accelerometer data during data processing.

### Data processing

The PALMS is a web-based application capable of combining activity data (e.g., accelerometers) with location data (GPS). PALMS aggregated and processed the accelerometer data to provide values for MVPA using 15 s cut points (39, 40). Evenson cut points (39) were used in this study with 574 counts per 15 s as threshold for MVPA. Continuous periods of 60 min of zero values were classified as non-wear time and removed (33, 41). PALMS processed the GPS data by identifying invalid data points using extreme speed or extreme changes in distance and elevation, and replaced invalid points by imputing data from the last known valid point, for up to 10 min. Using algorithms that utilize SNR PALMS categorized epochs as occurring indoors or outdoors. For this study, PALMS marked the locations as outdoor when the total SNR of all satellites in view exceeded a threshold of 250 (26, 29). Furthermore, PALMS identified and categorized trips (defined as a continuous period of movement of at least 3 min, allowing for stationary periods of maximum 5 min) into three modes: walking, bicycling, and vehicle (29). Processed GPS data were then matched to the accelerometer data in 15 s epochs, forming a PALMS dataset. The PALMS dataset consisted of 15 s accelerometer epochs with the following information appended: location (GPS coordinates: latitude and longitude), activity intensity (MVPA), outdoor (yes, no), and trip mode (walk, bicycle, and vehicle). In case no GPS signal was available the accelerometer epochs were retained to calculate the daily physical activity variables. The PALMS dataset is very rich in information and no available data management systems were able to handle the data load, or the specific requirements developed to obtain high quality, precise context-specific measures. Therefore, a purpose-built PostgreSQL database was developed. The PostgreSQL database was set-up to combine PALMS datasets with data from participant dairies, class timetables, and location data from a Geographical Information System (GIS, ArcGIS 10.1) to compute variables for days and context-specific settings. The context-specific settings included were the active living domains: leisure, school (scheduled school hours) (42), transport, and home (43) and a range of subdomains within these domains reflecting places where children and adolescents can be involved in MVPA. The subdomains constituting leisure were: school grounds (outside scheduled school hours) (44), clubs (after school programs), sports facilities (45), playgrounds (46, 47), urban green space (19, 20), shopping centers, and “other places.” The Municipality of Copenhagen provided the addresses of public schools, clubs, sports facilities, and playgrounds, enabling a manual digitizing of school grounds, clubs, sports facilities, and playgrounds in GIS. All urban green spaces were available from the Danish Geodata Agency. Major indoor shopping centers were identified online, and manually digitalized in GIS. Epochs not categorized as school, home, transport, or other leisure subdomains were categorized as other places. Epochs were assigned to school, recess, or PE according to the school schedule, adjusting for individual variations based on the individual student diary, and total recess and PE were then assessed within the school domain. All epochs classified by PALMS as trips, and not part of any other domain, constituted the transport domain, and PALMS trips were dichotomized into active (walking and biking) and passive (vehicle) transport. All students’ primary addresses were geocoded and each home was digitalized manually in GIS to constitute the home domain. A house was in GIS defined as the parcel, while an apartment was defined as the building and adjacent outdoor area. No subdomains were defined within the home domain. A 10-m Euclidian buffer was applied to all GIS derived domains and subdomains to account for signal and location errors. The database applied a hierarchical process to ensure an epoch could only be assigned to one context. All epochs belonging to the school domain were categorized first, followed by epochs belonging to first the home and then the leisure domain. Epochs belonging to a trip were then assigned to the transport domain, while the left over epochs finally were assigned to the leisure subdomain “other places”. The data were aggregated on an individual level by day, domain, and subdomain in the database before being imported into a statistical software package for further analyses.

### Outcome measures

For weekdays, 4 domains, and 11 subdomains, five daily context-specific outcome measures were calculated and used in this study: minutes of outdoor time, the proportion of time spent outdoors, minutes of outdoor MVPA, the proportion of MVPA spent outdoor, and MVPA. Proportion of time spent outdoors was calculated as minutes of outdoor time out of (wear) time during the average weekday or context-specific setting and hence expresses the proportion of time accumulated in a context that was outdoors. The proportion of MVPA spent outdoors was calculated as minutes of outdoor MVPA in a weekday, domain or subdomain out of total minutes of MVPA accumulated in the day, domain or subdomain and hence expresses the percentage of how much of all MVPA accumulated during the day, domain, and subdomain that is occurring outdoors. These measures were included to account for potential differences in movement patterns among groups, e.g., boys and girls may spend equal amount of time in a context but one part may spend double the amount of outdoor time.

### Data analyses

All analyses were performed using STATA SE12. Descriptive statistics were used to assess age, gender, and BMI by means of frequency distribution (%), and mean and standard deviation. Median and inter quartile ranges (IQR) were used to describe minutes of daily MVPA and wear time as these variables were not normally distributed. Univariable analyses were performed to evaluate the association between the two age groups and between boys and girls using a chi-square, *t*-test, or Wilcoxon rank-sum test. As the majority of outcome measures were not normally distributed, median and IQR were used to describe four of the outcome measures: outdoor times, the proportion of time spent outdoors, outdoor MVPA and the proportion of MVPA spent outdoors in total, domains and subdomains by gender, age, and totals. Multilevel analyses were used to provide results on age and gender differences. All models included students within school, further adjusting for BMI, number of valid weekdays (1–4) and daily wear time (models based on total days) or time in overall domain being investigated, e.g., the subdomain playground was adjusted for time in the leisure domain. Each model accounted for the nested nature of children within schools (48) by including school as a fixed effect. Models with a non-normal distribution of the residuals (49 out of 64 models) had their outcome transformed to fulfill the model assumptions, 22 by square root, 17 by log, 8 by *x*^2^, and 2 by *x*^3^ transformation. For the 17 log transformed models, zeros were replaced with a small number (0.03125 corresponding to half the value of the lowest non-zero number across the models) before transformation. The transformed model *p*-value and the untransformed model coefficient for age and gender differences are shown for ease of interpretation. Age and gender interactions were found in 15 out of 64 models (significance level *p* = 0.05). The interaction *p*-value and the significant gender and age subgroup differences are presented separately. Multilevel analyses were used to provide unadjusted and adjusted results on the association between MVPA and outdoor time during the total day, domains, and subdomains. All the adjusted models accounted for the nested nature of children within schools by including school as a fixed effect, further adjusting for age, gender, BMI, number of valid weekdays (1–4) and registered time in day (models based on total days), or time in overall domain being investigated. All models were tested for interactions between outdoor time, age, and/or gender (significance level *p* = 0.05) to investigate if the association was persistent across age and gender groups. An interaction was present in 7 out of 16 contexts; however, the pattern of the association (i.e., the size of the *p*-value) was the same across all subgroups with four exceptions. For ease of interpretation, the results from the untransformed models showing totals rather than subgroups are shown, while the text specifies the four subgroup exceptions.

## Results

### Participants

Table 1 shows characteristic for the study participants (*n* = 170). On average, the participants had a daily median of 12.9 h (IQR, 11.7–13.6) of combined accelerometer and GPS data and a mean of 2.7 valid days (SD 1.1) out of 4 possible. Boys compared to girls had more minutes of daily MVPA (82.8 versus 61.2 min, *p* < 0.001). As expected, BMI was greater among adolescents compared to children (*p* < 0.05).

**Table 1:** **Study participants (*n* = 170)**.

	Girls	Boys	Children	Adolescents	Total
Population (%)	87 (51.2)	83 (48.8)	129 (75.9)	41 (24.1)	170 (100)
Mean age (SD)	12.9 (1.2)	12.8 (1.0)	12.4 (0.7)***	14.2 (0.8)***	12.8 (1.1)
Mean BMI (SD)^a^	18.1 (2.8)	18.6 (3.2)	18.1 (3.1)*	19.4 (2.8)*	18.4 (3.0)
Mean valid days (SD)	2.7 (1.1)	2.6 (1.1)	2.7 (1.1)	2.4 (0.9)	2.7 (1.1)
Median daily minutes MVPA (IQR)	61.2 (46.7–75.8)***	82.8 (58.4–99.1)***	69.5 (53.5–91.9)	58.3 (46.8–85.6)	68.4 (52.0–91.8)
Median daily hours combined data (IQR)	13.0 (11.9–13.6)	12.7 (11.5–13.6)	12.9 (11.9–13.6)	13.0 (10.9–13.7)	12.9 (11.7–13.6)

*Significant difference *p* < 0.05.

***Significant difference *p* < 0.001.

*^a^*n* = 156*.

*BMI, body mass index; IQR, inter quartile range; MVPA, moderate to vigorous physical activity; SD, standard deviation*.

### Time outdoor pattern

Table 2 shows the daily median minutes of outdoor time (minutes, IQR) in total, domains, and subdomains by gender and age, and age and gender differences assessed in multilevel analyses. Across all groups, the majority of outdoor time was accumulated during the school hours, followed by leisure time, transport, and home. However, the pattern was less clear among adolescents.

**Table 2:** **Daily outdoor time in total/domain/subdomain by age and gender (*n* = 91–170) and adjusted age and gender differences (*n* = 81–156)**.

	Unadjusted median minutes (IQR)						Adjusted differences, minutes				
	Girls	Boys	Children	Adolescent	Total	*n*	Gender		Age		*n*
							Reference: girls		Reference: children	
							Coefficient	*p*-Value	Coefficient	*p*-Value	
Total	194.5 (129.1–259.3)	226.7 (184.8–291.3)	226.5 (175.0–284.5)	172.6 (111.7–225.3)	215.1 (157.7–278.5)	170	**34.9**	**0.001**	*****−***29.3**	**0.037**	156
Leisure	45.0 (21.2–73.0)	71.9 (33.3–110.5)	62.9 (32.0–107.5)	33.4 (23.1–73.0)	56.6 (29.0–96.8)	170	**21.3**	**0.000**	1.6	0.91	156
School grounds	9.3 (3.9–26.8)	15.3 (6.3–28.9)	15.3 (6.8–33.0)	4.1 (2.7–9.3)	11.7 (4.6–28.5)	170	3.9	0.12	*****−***8.7**	**0.004**	156
Clubs	0.0 (0.0–0.5)	0.0 (0.0–0.6)	0.0 (0.0–0.5)	0.0 (0.0–0.6)	0.0 (0.0–0.6)	170	−4.5	0.41	−4.0	0.49	156
Sports facilities	0.0 (0.0–0.6)	0.1 (0.0–12.8)	0.0 (0.0–1.0)	0.1 (0.0–29.3)	0.0 (0.0–2.9)	170	**8.1**	**0.002**	**9.7**	**0.001**	156
Playgrounds	0.0 (0.0–0.4)	0.3 (0.0–0.9)	0.0 (0.0–0.8)	0.2 (0.0–0.4)	0.0 (0.0–0.7)	170	0.1	0.10	−0.4	0.46	156
Urban green space	4.7 (2.0–9.8)	6.6 (2.0–11.8)	5.0 (2.0–10.8)	5.8 (2.8–9.3)	5.2 (2.0–10.3)	170	0.8	0.23	1.5	0.60	156
Shopping center	0.0 (0.0–0.1)	0.0 (0.0–0.0)	0.0 (0.0–0.0)	0.0 (0.0–0.8)	0.0 (0.0–0.1)	170	−0.1	0.43	**0.3**	**0.000**	156
Other places	10.5 (4.7–23.5)	23.3 (8.9–36.3)	16.1 (7.2–33.8)	11.5 (3.8–25.1)	14.8 (7.0–32.3)	170	**12.8**	**0.000**	3.2	0.44	156
School	80.6 (45.6–114.4)	91.5 (62.8–131.5)	96.5 (65.2–138.3)	44.5 (28.1–84.4)	87.0 (53.5–126.8)	168	0.4	0.90	*****−***36.4**	**0.000**	154
Recess	27.7 (18.4–34.7)	29.8 (21.3–40.3)	30.5 (23.2–39.0)	17.3 (12.9–28.8)	28.7 (19.4–36.4)	164	2.5	0.15	*****−***11.8**	**0.000**	151
PE	21.9 (6.0–65.5)	49.3 (16.5–85.3)	52.8 (8.4–86.3)	19.5 (13.3–44.3)	34.5 (11.0–83.8)	91	9.7	0.08	−11.2	0.92	81
Transport	23.7 (14.0–36.3)	29.0 (18.4–43.5)	24.8 (14.8–36.3)	30.2 (21.0–49.3)	25.9 (15.5–38.3)	170	**4.1**	**0.004**	**1.6**	**0.049**	156
Active	15.9 (8.6–25.4)	20.9 (11.5–31.1)	18.8 (9.8–26.3)	21.0 (10.5–31.3)	19.0 (10.5–28.5)	170	**3.1**	**0.019**	−1.5	0.83	156
Passive	4.2 (0.0–15.5)	3.0 (0.0–14.3)	2.1 (0.0–11.4)	10.0 (0.0–19.5)	3.8 (0.0–14.3)	170	0.4	0.48	2.1	0.13	156
Home	17.8 (4.8–51.4)	14.3 (4.8–51.5)	16.6 (4.4–49.7)	14.3 (5.3–55.4)	16.6 (4.8–51.4)	170	−4.5	0.99	6.3	0.80	156

*Bold: significance level at *p* < 0.05*.

*Differences in age (or gender) were estimated using multilevel analyses adjusted for differences in gender (or age), BMI, number of valid days, and time in domain. School included as fixed effect to account for clustering of students within school*.

*IQR, inter quartile range; MVPA, moderate to vigorous physical activity; PE, physical education*.

While boys were outdoors 226.7 min per day, girls were outdoors 194.5 min per day (*p* < 0.05) (Table 2). This difference was predominantly due to a difference during leisure, where boys were outdoors 71.9 min and girls were outdoors only 45.0 min (*p* < 0.001). Within leisure, boys spent more time outdoors when in sports facilities and other places (all *p* < 0.05). Boys also spent more time outdoors when in transport (*p* < 0.05). There was no gender difference in the time spent outdoors during school or home.

Children were outdoors a median of 226.5 min per day and adolescents 172.6 min per day (*p* < 0.05) (Table 2). This difference originated predominantly from a difference during school hours, were children compared to adolescents had almost the double amount of outdoor time (96.5 versus 44.5 min, *p* < 0.001). Children also spent more time outdoors when at school grounds outside school hours (15.3 versus 4.1 min, *p* < 0.05) but spent fewer minutes outdoors than adolescence during transport (24.8 versus 30.2 min, *p* < 0.05).

Table 3 shows the daily median proportion of time spent outdoors (%, IQR) in total, domains, and subdomains by gender and age, and age and gender differences assessed in multilevel analyses. Boys spent a greater proportion of their time during a day being outdoors: boys were outdoors 29.0% of the day while girls were outdoors 22.2% of the day (*p* < 0.05). The proportion of time spent outdoors when in a particular domain or subdomain varied; with home being the place where the lowest proportion of time was spent outdoors (girls 8.5%, boys 7.9%) and playgrounds had the highest proportion (girls 87.5%, boys 99.8%). Besides spending a larger proportion of time outdoors in leisure, sports facilities, other places, and in transport, boys compared to girls also spent a greater proportion of their time outdoors in school grounds, during recess, and PE. No gender difference was detected in the proportion of time spent outdoors when in active transport, despite boys spending significant more minutes outdoors in active transport.

**Table 3:** **Daily proportion of time in total/domain/subdomain that is spent outdoor by age and gender (*n* = 53–170) and adjusted age and gender differences (*n* = 48–156)**.

	Unadjusted median % (IQR)						Adjusted differences, %				
	Girls	Boys	Children	Adolescent	Total	*n*	Gender		Age		*n*
							Reference: girls		Reference: children	
							Coefficient	*p*-Value	Coefficient	*p*-Value
Total	22.2 (16.0–30.7)	29.0 (22.7–36.8)	27.3 (20.5–35.3)	20.6 (13.9–27.2)	25.7 (19.2–34.5)	170	**4.3**	**0.001**	−3.2	0.05	156
Leisure	18.7 (9.9–30.7)	26.0 (16.3–41.9)	23.3 (14.2–33)	21.1 (10.2–33.7)	22.6 (12.5–33.2)	170	**7.7**	**0.001**	1.0	0.92	156
School grounds	41.7 (27.8–58.5)	63.0 (48.0–73.2)	55.7 (36.2–70.5)	32.2 (22.6–56.1)	51.7 (30.9–68.3)	167	**8.7**	**0.004**	**−8.0**	**0.043**	154
Clubs	37.1 (1.6–72.8)	53.9 (14.5–100.0)	47.4 (2.9–97.5)	33.3 (11.1–72.2)	46.0 (4.0–95.1)	91	14.9	0.09	−3.7	0.60	84
Sports facilities	54.3 (1.4–96.4)	92.7 (7.5–100.0)	66.7 (0.0–100.0)	93.2 (50.0–100.0)	76.3 (6.3–100.0)	109	**15.4**	**0.017**	22.2	0.10	102
Playgrounds	87.5 (38.1–100.0)	99.8 (50.0–100.0)	94.0 (36.4–100.0)	95.7 (72.7–100.0)	94.0 (44.9–100.0)	88	13.9	0.15	9.8	0.37	82
Urban green space	66.6 (38.5–78.9)	71.3 (41.2–92.2)	67.4 (33.3–88.8)	77.1 (57.6–86.6)	68.5 (38.9–88.2)	167	8.9	0.05	5.5	0.35	154
Shopping center	41.6 (11.5–60.0)	15.5 (1.7–74.2)	24.0 (0.8–55.2)	50.0 (14.3–75.0)	33.3 (3.7–60.3)	53	−0.5	0.96	10.4	0.40	48
Other places	7.2 (3.2–15.9)	11.5 (5.6–22.5)	9.8 (4.8–16.7)	8.2 (3.3–21.0)	9.3 (4.2–17.2)	170	**6.8**	**0.000**	3.7	0.23	156
School	25.0 (13.6–36.8)	30.7 (17.3–45.9)	31.1 (19.4–46.4)	13.8 (9.2–29.5)	28.3 (15.4–43.7)	168	0.0	0.84	**−12.7**	**0.000**	154
Recess	43.6 (28.8–62.6)	55.9 (41.6–73.2)	57.4 (41.7–72.2)	26.0 (20.2–42.7)	49.8 (33.8–66.7)	164	**5.1**	**0.030**	**−23.6**	**0.000**	151
PE^5^	32.8 (6.0–73)	59.9 (21.6–95.0)	71.3 (9.8–96.1)	21.8 (14.0–52.5)	52.0 (12.0–93.3)	91	**14.2**	**0.030**	−8.9	0.84	81
Transport	73.8 (58.6–85.8)	76.5 (65.6–89.4)	76.5 (62.5–87.3)	74.5 (62.9–86.9)	75.0 (62.6–87.3)	170	**7.9**	**0.003**	3.0	0.39	156
Active	81.1 (68.5–92.9)	83.7 (69.2–91.3)	81.1 (67.0–92.6)	83.0 (74.4–92.6)	81.2 (68.9–92.6)	168	2.9	0.34	4.9	0.24	154
Passive	52.1 (38.7–65.5)	60.4 (47.6–79.4)	53.4 (38.7–69.5)	59.4 (48.3–77.5)	55.3 (40.9–72.5)	111	**10.4**	**0.011**	4.9	0.34	101
Home	8.5 (2.9–22.6)	7.9 (3.3–26.1)	8.9 (3.2–23.1)	7.8 (2.8–22.6)	8.5 (3.1–22.9)	169	2.9	0.987	−1.4	0.937	155

*Bold: significance level at *p* < 0.05*.

*Differences in age (or gender) were estimated using multilevel analyses adjusted for differences in gender (or age), BMI, number of valid days, and time in domain. School included as fixed effect to account for clustering of students within school*.

*IQR, inter quartile range; MVPA, moderate to vigorous physical activity; PE, physical education*.

A trend of an overall age difference in the proportion of time spent outdoors during the total day was detected with children being outdoors 27.3% of the day and adolescents 20.6% of the day (*p* = 0.05) (Table 3). Children compared to adolescents accumulated a larger proportion of their MVPA at school grounds (*p* < 0.05), during school hours (*p* < 0.001), and during recess (*p* < 0.001).

An analysis of age and gender interactions further revealed that adolescent girls had less daily outdoor time and spent a lower proportion of time outdoor on school grounds and at other places than child girls and boys (Table 6). Girl children also spent a smaller proportion of time in other places than boys. Adolescent boys spent more leisure time outdoor and spent a larger proportion of time outdoor than girls and child boys. Child boys spent more leisure time outdoor and spent a larger proportion of outdoor time than adolescent girls. Adolescent boys spent more time outdoors in urban green space than adolescent girls.

### Outdoor MVPA pattern

Table 4 shows the daily median minutes of outdoor MVPA time (outdoor MVPA minutes, IQR) in total, domains, and subdomains by gender and age and age and gender differences assessed in multilevel analyses. Girls accumulated a daily median of 42.3 min of outdoor MVPA, while boys accumulated a daily median of 61.8 min of outdoor MVPA (*p* < 0.001). In 10 out of the 15 investigated contexts, boys compared to girls accumulated more minutes of outdoor MVPA, with no gender differences present in clubs, playgrounds, urban green space, shopping centers, and passive transport (*p* > 0.05).

**Table 4:** **Daily outdoor MVPA in total/domain/subdomain by age and gender (*n* = 91–170) and adjusted age and gender differences (*n* = 81–156)**.

	Unadjusted median minutes (IQR)						Adjusted differences, minutes				
	Girls	Boys	Children	Adolescent	Total	*n*	Gender		Age		*n*
							Reference: girls		Reference: children	
							Coefficient	*p*-Value	Coefficient	*p*-Value
Total	42.3 (25.3–52.3)	61.8 (41.0–76.0)	50.1 (36.5–65.5)	38.4 (23.8–66.3)	48.1 (34.3–65.7)	170	**24.3**	**0.000**	1.0	0.57	156
Leisure time	7 (4.1–14.5)	14.8 (5.8–26.8)	12.1 (5.0–21.6)	7.8 (4.3–19.6)	11.1 (4.7–21.3)	170	**8.8**	**0.000**	3.7	0.07	156
School grounds	1.8 (0.9–5.0)	3.5 (1.5–9.0)	3.4 (1.5–7.1)	1.4 (0.8–2.2)	2.5 (1.2–6.2)	170	**3.6**	**0.002**	−0.8	0.06	156
Clubs	0.0 (0.0–0.3)	0.0 (0.0–0.2)	0.0 (0.0–0.3)	0.0 (0.0–0.1)	0.0 (0.0–0.2)	170	−0.4	0.22	−0.4	0.61	156
Sports facilities	0.0 (0.0–0.1)	0.0 (0.0–4.5)	0.0 (0.0–0.4)	0.0 (0.0–5.1)	0.0 (0.0–0.7)	170	**2.1**	**0.000**	**2.4**	**0.004**	156
Playgrounds	0.0 (0.0–0.3)	0.0 (0.0–0.4)	0.0 (0.0–0.4)	0.0 (0.0–0.3)	0.0 (0.0–0.3)	170	0.2	0.06	0.0	0.52	156
Urban green space	1.5 (0.8–3.8)	1.8 (0.8–4.9)	1.5 (0.6–3.6)	2.3 (1.1–4.4)	1.8 (0.8–3.8)	170	0.9	0.14	1.2	0.06	156
Shopping center	0.0 (0.0–0.0)	0.0 (0.0–0.0)	0.0 (0.0–0.0)	0.0 (0.0–0.1)	0.0 (0.0–0.0)	170	0.0	0.19	**0.0**	**0.006**	156
Other places	0.8 (0.3–2.1)	1.3 (0.5–3.9)	1.0 (0.5–2.5)	1.3 (0.3–2.6)	1.0 (0.4–2.5)	170	**2.5**	**0.001**	1.2	0.09	156
School	13.4 (8.7–19.9)	21.5 (12.1–30.4)	18.9 (12.3–28.2)	7.7 (4.0–17.8)	16.7 (10.2–27.4)	168	**7.1**	**0.000**	**−10.1**	**0.000**	154
Recess	4.6 (2.7–7.3)	6.6 (4.3–11.1)	6.3 (4.2–9.3)	2.9 (1.4–6.7)	5.5 (3.5–8.9)	164	**2.8**	**0.000**	**−3.4**	**0.000**	151
PE	8.9 (1.3–17.5)	21.5 (5.0–34)	13.9 (1.3–32.1)	8.3 (4.3–20.4)	11.3 (1.8–25.0)	91	**9.5**	**0.001**	−1.6	0.68	81
Transport	10.1 (3.6–16.7)	11.9 (5.8–19.6)	10.3 (4.8–15.8)	15.9 (5.2–20.1)	10.9 (4.8–17.5)	170	**2.3**	**0.039**	3.0	0.09	156
Active	6.8 (3.0–13)	9.3 (5.3–15.1)	8.1 (3.4–13.3)	9.1 (4.3–16.4)	8.3 (3.5–14.6)	170	**2.3**	**0.020**	1.2	0.42	156
Passive	0.3 (0.0–3.7)	0.6 (0.0–4.7)	0.3 (0.0–3.2)	2.3 (0.0–7.3)	0.4 (0.0–4.2)	170	0.0	0.87	**1.7**	**0.002**	156
Home	1.8 (0.6–5.3)	2.5 (0.4–9.3)	1.8 (0.5–6.4)	3.3 (1.0–9.6)	2.3 (0.5–6.8)	170	**5.0**	**0.035**	−0.4	0.32	156

*Bold: significance level at *p* < 0.05*.

*Differences in age (or gender) were estimated using multilevel analyses adjusted for differences in gender (or age), BMI, number of valid days, and time in domain. School included as fixed effect to account for clustering of students within school*.

*IQR, inter quartile range; MVPA, moderate to vigorous physical activity; PE, physical education*.

Among children and adolescents, no overall difference was found in how many minutes of outdoor MVPA were accumulated during the whole day (*p* > 0.1) (Table 4). Children compared to adolescents had more outdoor MVPA during school hours and recess (*p* < 0.001) while adolescent compared to children had more outdoor MVPA at sport facilities, shopping centers, and passive transport (*p* < 0.05).

In the analyses investigating the proportion of MVPA occurring outdoors during the day and in different contexts (Table 5), a significant gender difference was detected overall and in 6 out of the 15 investigated contexts. Boys accumulating a larger proportion of their MVPA outdoors when in leisure overall, in school grounds, sports facilities, playgrounds, school, and PE. During the total day, 73.8% of boys MVPA was spent outdoors with girls spending 65.3% of their MVPA outdoors (*p* < 0.001). No overall difference was found between children and adolescents in the proportion of daily MVPA that was spent outdoors, but children spent a larger proportion of their MVPA outdoors during school hours and recess (*p* < 0.001).

**Table 5:** **Proportion of daily MVPA that is spent outdoor in total/domain/subdomain by age and gender (*n* = 34–170) and adjusted age and gender differences (*n* = 32–156)**.

	Unadjusted median % (IQR)						Adjusted differences, %				
	Girls	Boys	Children	Adolescent	Total	*n*	Gender		Age		*n*
							Reference: girls		Reference: children	
							Coefficient	*p*-Value	Coefficient	*p*-Value
Total	65.3 (55.9–71.4)	73.8 (68.6–78.4)	70.0 (63.5–76.5)	67.4 (52.1–74.7)	69.9 (62.5–76.1)	170	**7.7**	**0.000**	−5.5	0.06	156
Leisure time	55.6 (41.7–67.0)	63.3 (48.7–76.1)	61.2 (45.1–70.6)	62.5 (46.3–72.2)	61.3 (45.1–71.6)	170	**10.0**	**0.000**	3.3	0.47	156
School grounds	59.8 (46.7–75.0)	72.6 (60.3–84.6)	68.0 (56.3–79.7)	64.3 (46.7–82.1)	66.9 (50.8–80.5)	167	**10.1**	**0.001**	0.5	0.91	154
Clubs	100.0 (75.6–100.0)	84.8 (33.3–100.0)	95.8 (55.9–100.0)	100.0 (50.0–100.0)	100.0 (50.0–100.0)	65	−14.3	0.11	−24.6	0.11	61
Sports facilities	97.5 (45.0–100.0)	100.0 (99.4–100.0)	100.0 (50.0–100.0)	100.0 (99.4–100.0)	100.0 (87.5–100.0)	70	**24.0**	**0.000**	17.5	0.10	68
Playgrounds	100.0 (70.0–100.0)	100.0 (97.4–100.0)	100.0 (84.5–100.0)	100.0 (87.5–100.0)	100.0 (87.5–100.0)	73	**9.0**	**0.048**	−12.0	0.17	67
Urban green space	96.3 (82.9–100.0)	98.7 (81.3–100.0)	97.0 (78.7–100.0)	97.5 (85.7–100.0)	97.3 (81.3–100.0)	158	0.5	0.56	2.1	0.62	148
Shopping center	61.0 (27.4–100.0)	45.0 (22.2–100.0)	62.5 (37.9–100.0)	33.3 (22.2–100.0)	54.8 (22.2–100.0)	34	1.6	0.90	11.9	0.41	32
Other places	24.9 (9.2–36.3)	26.8 (11.6–45.1)	25.6 (10.4–40.4)	26.7 (5.3–36.7)	26.2 (10.2–40.0)	169	5.6	0.07	1.7	0.52	155
School	62.0 (51.6–70.4)	72.2 (63.3–78.4)	67.3 (58.3–77.9)	49.7 (31.2–69.4)	66.0 (53.7–76.6)	168	**5.8**	**0.014**	**−17.4**	**0.000**	154
Recess	67.4 (57.0–79.3)	78.3 (61.9–83.4)	76.4 (63.0–82.7)	57.6 (36.1–76.4)	73.8 (59.2–81.5)	164	4.2	0.09	**−22.7**	**0.000**	151
PE	52.2 (6.0–91.9)	85.3 (36.7–98.9)	91.9 (15.7–98.7)	59.6 (29.4–85.3)	81.4 (20.0–98.1)	89	**13.7**	**0.033**	5.7	0.11	79
Transport	89.5 (83.3–96.5)	91.4 (84.0–96.0)	89.4 (82.4–96.3)	91.7 (88.1–96.1)	90.1 (83.4–96.1)	168	2.3	0.25	1.5	0.56	155
Active	91.6 (83.9–97.9)	92.7 (84.6–97.1)	91.7 (83.1–97.4)	93.4 (87.6–97.5)	92.0 (84.0–97.4)	167	1.6	0.46	2.8	0.32	154
Passive	91.0 (77.4–100.0)	91.1 (78.8–98.4)	89.5 (77.3–100.0)	94.0 (83.3–98.7)	91.0 (77.4–100.0)	104	0.8	0.58	2.5	0.72	94
Home	38.1 (16.7–61.6)	36.9 (23.1–64.0)	36.4 (16.3–61.0)	52.8 (24.0–66.7)	37.1 (20.3–63.2)	169	3.4	0.39	1.8	0.72	155

*Bold: significance level at *p* < 0.05*.

*Differences in age (or gender) were estimated using multilevel analyses adjusted for differences in gender (or age), BMI, number of valid days, and time in domain. School included as fixed effect to account for clustering of students within school*.

*IQR, inter quartile range; MVPA, moderate to vigorous physical activity; PE, physical education*.

When in transport, clubs, sport facilities, playgrounds, urban green space, and in recess a high proportion of MVPA took place outdoors for both boys and girls, and children and adolescents (84.8–100%). Boys and children also accumulated a large proportion of their MVPA outdoors when in PE (boys 85.3%, children 91.9%) (Table 5).

Adolescent boys accumulated more outdoor MVPA minutes in urban green space and home than child boys and girls (Table 6). Adolescent boys also spent a larger proportion of their MVPA outdoors during leisure, in school grounds, and other places. Adolescent boys spent a larger proportion of their MVPA outdoors when at home compared to adolescent girls. Child boys had more minutes of outdoor MVPA compared to adolescent boys and girls. Child girls spent a lower proportion of their MVPA outdoors while at sports facilities compared to adolescent girls and boys.

**Table 6:** **Gender and age interactions by outdoor time, proportion of time spent outdoor, outdoor MVPA and proportion of MVPA spent outdoors in total, domains, and subdomains**.

	Outdoor time		% Time spent outdoors		Outdoor MVPA		% Of MVPA spent outdoors	
	*p*-Value^a^	Significant interaction group differences	*p*-Value^a^	Significant interaction group differences	*p*-Value^a^	Significant interaction group differences	*p*-Value^a^	Significant interaction group differences
Total outdoor time	0.003	GA < GC, BA, BC	0.002	GA < GC, BA, BC				
Leisure	0.003	BA > GA, GC, BC > GA	0.004	BA > GA, GC, BC > GA			0.017	BA > BC, GA, GC
School grounds			0.037	GA < GC, BA, BC			0.003	BA > GC, GA, BC > GA
Clubs								
Sports facilities							0.012	GC < GA, BA, BC
Playgrounds								
Urban green space	0.045	BA > GA			0.017	BA > GC, GA, BC		
Shopping center								
Other places			0.040	GA < BA, BC, GC < BC, BA			0.036	BA > BC, GC, GA
School								
Recess								
PE					0.008	BC > BA, GA, GC		
Transport								
Active								
Passive								
Home					0.021	BA > BC, GA, GC	0.044	BA > GA

*^a^Interaction *p*-value in multilevel analyses adjusted for BMI, number of valid days, and time in domain. School included as fixed effect to account for clustering of children within school*.

*GC, girl child (*n* = 60); GA, girl adolescent (*n* = 27); BC, boy child (*n* = 69); BA, boy adolescent (*n* = 14)*.

*IQR, inter quartile range; MVPA, moderate to vigorous physical activity; PE, physical education*.

### Time outdoor and MVPA

In multilevel analyses, time spent outdoors (hours) was a significant predictor of MVPA (minutes) both in unadjusted models and in models adjusted for potential confounders (gender, age, BMI, number of valid days, time in day, or overall domain) (Table 7). Models were run for days, domains, and subdomains to investigate if the association varied by context, but a consistent relationship was found throughout the day (all *p* < 0.001) with only four exceptions detected in supplementary analyses investigating interactions between outdoor time, gender, and age (data not shown). No association was found between outdoor time and MVPA for child boys when at shopping centers (*p* > 0.1) and in transport (*p* > 0.1). Also a weaker association was found for adolescent girls when in transport (*p* = 0.05) or at home (*p* = 0.06). During the course of the whole day, a 1-h increase in outdoor time was associated with 9.9 more minutes of MVPA. An association was also found for contexts in leisure time where a 1-h increase in outdoor time was associated with an increase of 23.5 more minutes of MVPA in school grounds, 20.2 more minutes of MVPA in urban green space, and 18.6 more minutes of MVPA when at sports facilities. One more hour of outdoor time during active transport was associated with 28.5 more minutes of MVPA (all *p* < 0.000).

**Table 7:** **Association between time outdoors (hours) and MVPA (minutes) in total weekdays, domains, and subdomains**.

	Model 1			Model 2		
	Coef.	*p*	95% CI	Coef.	*p*	95% CI
Total day	10.9	<0.001	8.0–13.8	9.8	<0.001	6.9–12.8
Leisure time	14.8	<0.001	12.3–17.4	13.5	<0.001	10.7–16.4
School grounds	21.7	<0.001	19.5–23.9	23.5	<0.001	21.1–25.9
Clubs	10.7	<0.001	9.0–12.4	11.2	<0.001	9.4–13.0
Sports facilities	18.0	<0.001	16.4–19.6	18.6	<0.001	16.8–20.3
Playgrounds	16.3	<0.001	15.5–17.1	16.4	<0.001	15.6–17.3
Urban green space	20.2	<0.001	18.5–21.8	20.2	<0.001	18.4–21.9
Shopping center	15.9	<0.001	12.5–19.3	17.3	<0.001	13.7–20.9
Other places	7.3	<0.001	4.9–9.7	5.5	<0.001	2.9–8.1
School	9.0	<0.001	6.5–11.4	8.3	<0.001	5.8–10.8
Recess	11.6	<0.001	7.8–15.4	8.5	<0.001	4.3–12.6
PE	13.1	<0.001	8.2–18.0	10.9	<0.001	5.9–15.9
Transport	21.2	<0.001	17.7–24.6	15.0	0.002	5.6–24.4
Active	29.3	<0.001	25.4–33.2	28.5	<0.001	23.5–33.6
Passive	10.6	<0.001	8.6–12.6	6.5	<0.001	3.6–9.3
Home	10.1	<0.001	8.3–11.9	9.1	<0.001	7.1–11.2

*Model 1: unadjusted multilevel analyses*.

*Model 2: multilevel analyses adjusted for age, gender, BMI, valid days, time in day/domain, and clustering of students within schools*.

*Coef., mean increase in minutes of MVPA associated with a 1-h increase in outdoor time*.

*IQR, inter quartile range; MVPA, moderate to vigorous physical activity; PE, physical education*.

## Discussion

This study investigated the volume and pattern of context-specific weekday outdoor time, outdoor MVPA, and the association between context-specific daily MVPA and outdoor time using combined accelerometer and GPS data for 170 children aged 11–16 years old. Four domains, 11 subdomains, and daily medians were assessed as context-specific measures and age and gender differences were investigated. A different pattern was found for boys and girls, as well as for children and adolescents. Girls compared to boys had fewer outdoors minutes and spent a lower proportion of their daily time outdoors overall and in the majority of investigated contexts. Girls compared to boys had fewer outdoor MVPA minutes during the day and in 11 contexts. A less consistent difference was found for the proportion of MVPA spent outdoors; gender differences were only detected in five contexts. During the total weekday, children compared to adolescents had more outdoor minutes (*p* < 0.05) while no difference in daily outdoor MVPA behavior was found. However, across all investigated outcomes a difference in behavior in the school context was detected, with children engaging in more outdoor MVPA and spending more time outdoors during school hours and within recess. Finally, it was found that outdoor time was a correlate for MVPA across the total day, all domains and subdomains.

Overall, 21.8–29.3% of time was spent outdoors, corresponding to approximately 3 h a day. Compared to other studies, even though the studies are not directly comparable as the methods used differ, it appears that the Danish children studied were spending more time outdoors than children included in studies from the UK (19, 25), Australia (2, 6), and Switzerland (49). This discrepancy could be due to outdoor time being measured differently; the Australian and Swiss studies relied on self-report data and the UK studies used a GPS device that assessed outdoor time differently from the present study. Another difference might be time of year when the data were collected as seasonality and weather conditions previously have been related to objectively assessed physical activity in children (50) and this association is likely to also apply to outdoor times.

In agreement with other studies, boys spent more time outdoors than girls (6, 25) and children spent more time outdoors compared to adolescents (49). The majority of outdoor time was for all groups occurring during school hours followed by leisure time. This study is one of the first to estimate the proportion of outdoor time occurring in domains during the day and further studies are needed to confirm this finding.

Between 62.3 and 71.0% of all total daily MVPA was accumulated outdoors. In the PEACH study, it was found that 26.4–35% of MVPA took place outdoors on weekdays outside the school hours (19). Even though not directly comparable, it seems like the present study population experienced a greater proportion of MVPA outdoors. Similar to the PEACH study, outdoor time in the present study was found to be a significant predictor of daily MVPA in all investigated contexts, and a 1-h increase in outdoor time was associated with almost 10 more minutes of MVPA per day. This study shows a stronger relationship between outdoor time and MVPA than a previous study using self-reported measures from parents (6). Here it was found that for every additional hour spent outdoors per week, MVPA increased by 27 min per week (almost 4 min per day) among 10–12 year old children. The stronger association found in this study may be due to self-reported measures from children or parents being imprecise, and the inclusion of GPS data may provide a more reliable estimate of actual outdoor time, and the accelerometer a more reliable estimate of physical activity. The overall level of daily MVPA among the participants in our study was also high, reaching a daily median of 67.3 min of MVPA. As this study is based on a cross-sectional sample it is not possible to conclude the causal direction of outdoor time and MVPA. Longitudinal studies are needed to establish a causal relationship between outdoor time and MVPA, but promoting outdoor time among the investigated group may have a range of health benefits beyond the association with physical activity. Modifiable characteristics in the neighborhood such as sidewalks, parallel or grouped parking places, traffic safety, and roundabouts have been associated with outdoor play (51), and parental concern about traffic safety has been associated with less time playing outdoors (49, 51). When planning urban renewal programs or new neighborhoods, factors that may be associated with increased or decreased outdoor time are important to consider.

No studies to date have investigated what is needed to obtain reliable estimates of children’s context-specific physical activity patterns based on the combination of accelerometers and GPS, and measurement decisions are relaying on recommendations for accelerometer studies. Future methodological studies are needed to investigate if and how the use of combined accelerometers and GPS should differ in the design from a study based solely on accelerometers. Experiences from this study indicate some specific areas that need careful consideration before embarking on research studies using combined accelerometer and GPS measurements. A larger drop-out was seen in this study compared to a similar project using only accelerometers conducted simultaneous (52). Both during data collection (fewer persons consented, more opted out or lost equipment during data collection, device-failure, e.g., software resulting in missing information on SNR), but also in the data analyses were it was evident that less rigid demands had to be applied to wear time and number of valid days to retain a reasonable study population. It may be reasonable to hypothesize that the inclusion of the GPS placed a greater burden on the participants, leading to less compliance with the study protocol. Therefore, it is recommended that studies using accelerometer and GPS should consider: (a) to oversample or (b) to ask participants to wear the equipment for a longer period of time. Solution: (a) implies either a longer data collection period or a greater pool of equipment while solution (b) again increases the participant burden. A method seen and recommended in accelerometer studies to increase the number of valid participants is to quickly download and screen data to see if a participant complied with the protocol. If not, they are asked to re-wear the accelerometer (53). Similar procedures for the GPS may also be feasible; however, it is time-consuming.

Using a combination of accelerometers and GPS to develop context-specific measures has a large potential to lead to new knowledge on physical activity, inform the development of new interventions, and perhaps later lead to new policies or recommendations for specific subgroups. Still, it is not an easy fix. The data collection is more complex, the recruitment of participants harder, and the data processing and complexity of the data is overwhelming. Practical issues of data storage capacity and run times of 2–3 weeks to generate variables in the purpose-built database for subsamples of 100 participants were a reality in this study, making the analyses time-consuming and labor-intensive. The development of PALMS is one major step toward a resource that can help researchers to start process their data. Integrating the methodology behind this study into PALMS has the potential to increase the number of studies investigating context-specific behaviors, as well as lead to greater conformity across studies, making comparisons possible, and hence perhaps increase knowledge on generalizable context-specific behaviors more quickly.

### Strengths and limitations

The use of PALMS to detect outdoor time is relatively novel and the algorithm used by PALMS has been validated in one study (26), however further validation may be needed. We found a large proportion of the participant’s time during school hours was spent outdoors. This may be a true finding but it may also be due to a problem with detecting outdoor time accurately at the schools included in this study. In many school buildings in Denmark, classrooms are situated along the outer walls with large windows. This combination may lead to a good satellite reception inside some classrooms, which could give some misclassification of indoor points being classified as outdoors. By definition, the proportion of time spent outdoors in contexts like playgrounds, urban green space, and active transport should have approximated 100% while we found averages ranging from 66.6 to 99.8%. Manual inspection of the data confirmed that some epochs taking place outdoors were misclassified as indoors due to the SNR under heavy tree canopies or close to tall buildings presenting as low as 65. PALMS requires the SNR to be above 250 before classifying an epoch as outdoors. The PALMS classifications employ an SNR threshold, and reducing it would affect accuracy overall. Tree canopies are known to affect SNR so it is difficult to envisage a solution based on the GPS data alone. Post processing matched green space with the GPS data may help but cannot be automated. The prevalence of this problem is not known, but the impact of this misclassification will be an underestimation of the association between outdoor time and MVPA.

Almost all participants on two out of four schools had to be excluded as their GPS did not record the information needed to estimate outdoor time. This loss of data was due to malfunctioning Qstarz software not encountered during a pilot study. When detected, the open source software bt747 (bt747.org) was used instead, which eradicated the problem. When using novel technologies and devices, errors will inevitably happen, introducing possible systematic errors. The error happening in this study limited the number of participants that could be included, however it is not likely that this introduced a systematic error as the excluded participants did not differ from the included participants on important background characteristics.

This study focused on urban Danish children and as other studies have found differences in the urban/suburban/rural physical activity patterns (15, 54) the results may not be generalizable to more rural children. Also the children were selected based on their school attendance in four schools situated close to each other as they were part of a larger natural experiment evaluating changes to one specific local neighborhood. As such, further studies are needed to confirm the generalizability of the results. Data were only collected during early fall and late spring, where daylight and overall weather conditions were quite similar, and therefore it is also not known if the results are valid during the winter months (16).

This study is one of the first studies to describe the daily context-specific outdoor time and outdoor MVPA patterns among school-children using objective measures during weekdays. Future studies should also consider examining the weekend pattern as different patterns of physical activity have been described between week and weekend days (17). Also future studies should investigate if the association found between outdoor time and MVPA is consistent across subgroups. Age and gender interactions were present in some domains or subdomains but we chose not to stratify the data by subgroups due to sample size limitations. The sample size also cautions interpretation of the interactions presented in this paper and these results may not be generalizable to other populations. Future studies should further investigate age and gender interactions present in context-specific behavior and also investigate if other subgroups, e.g., overweight/obese, high/low socio-economic position, have a distinct context-specific pattern. However, researchers must consider the increased complexity this adds in presenting the data.

## Conclusion

Different context-specific behaviors were found for gender and age, suggesting different strategies may be needed to promote physical activity among these groups. Studies using a combination of accelerometer and GPS devices are increasing in numbers as the need for context-specific physical activity patterns to inform effective health promotion is being acknowledged. Using novel technologies involves novel data processing methods and analytic strategies, and to promote a strong evidence base it is important that uniform methods are used, making it possible to compare results across studies and perhaps in the future to pool data to investigate country differences. This study proposed a domain based methodology expanded with a number of subdomains to assess the context-specific outdoor time and physical activity patterns among school-children and this methodology can easily be transferred to other populations.

## Author Contributions

Charlotte Demant Klinker conceived and coordinated the study, was responsible for its design, acquisition of data, data cleaning, statistical analyses, and drafted the manuscript. Jasper Schipperijn handled and processed the data, contributed to the acquisition of data, and data cleaning. Jens Troelsen conceived the study, and participated in its design. Jacqueline Kerr contributed with significant input to the outline of the manuscript and Annette Kjær Ersbøll came with statistical input. All authors revised the manuscript critically, and read and approved the final manuscript.

## Conflict of Interest Statement

The authors declare that the research was conducted in the absence of any commercial or financial relationships that could be construed as a potential conflict of interest.
